# Widening Access to Bayesian Problem Solving

**DOI:** 10.3389/fpsyg.2020.00660

**Published:** 2020-04-09

**Authors:** Nicole Cruz, Saoirse Connor Desai, Stephen Dewitt, Ulrike Hahn, David Lagnado, Alice Liefgreen, Kirsty Phillips, Toby Pilditch, Marko Tešić

**Affiliations:** ^1^Department of Psychological Sciences, Birkbeck, University of London, London, United Kingdom; ^2^Department of Psychology, City, University of London, London, United Kingdom; ^3^Department of Experimental Psychology, University College London, London, United Kingdom

**Keywords:** Bayesian networks, assistive software technology, reasoning, decision making, probabilistic

## Abstract

Bayesian reasoning and decision making is widely considered normative because it minimizes prediction error in a coherent way. However, it is often difficult to apply Bayesian principles to complex real world problems, which typically have many unknowns and interconnected variables. Bayesian network modeling techniques make it possible to model such problems and obtain precise predictions about the causal impact that changing the value of one variable may have on the values of other variables connected to it. But Bayesian modeling is itself complex, and has until now remained largely inaccessible to lay people. In a large scale lab experiment, we provide proof of principle that a Bayesian network modeling tool, adapted to provide basic training and guidance on the modeling process to beginners without requiring knowledge of the mathematical machinery working behind the scenes, significantly helps lay people find normative Bayesian solutions to complex problems, compared to generic training on probabilistic reasoning. We discuss the implications of this finding for the use of Bayesian network software tools in applied contexts such as security, medical, forensic, economic or environmental decision making.

## Theoretical Background

Most reasoning situations arguably take place under uncertainty: we cannot say for sure that the information from which we draw inferences is correct, but only believe it to a higher or lower degree (Evans and Over, 2013; Pfeifer, 2013; Gilio and Sanfilippo, 2014; Over and Cruz, 2019; Oaksford and Chater, 2020). Moreover, these uncertain pieces of information may be related to one another in intricate ways, so that it can quickly become difficult to foresee the implications that a change in our degree of belief in one piece of information may have on our degrees of belief in the others (Fernbach et al., 2010; Hadjichristidis et al., 2014; Rottman and Hastie, 2016; Bramley et al., 2017; Rehder and Waldmann, 2017).

But just like we can make use of tools like notepads and video recorders to aid our memory, there are tools that can help us navigate complex reasoning tasks in which we have to draw inferences from uncertain information. In particular, we can use probability theory to establish precise constraints between related degrees of belief (e.g., Gilio and Over, 2012; Politzer, 2016), and we can use Bayesian networks (BNs) to establish the precise implications of a change in the probability of one piece of information for the probability of other, related pieces of information (Pearl, 1988, 2000; Korb and Nicholson, 2011; Fenton and Neil, 2018).

Bayesian networks are graphical representations of probabilistic dependency relations between variables. Each variable is represented through a node, and arrows represent directed links from one node to another. Each node is associated with a probability table. The “parent” nodes in the network, which do not have arrows leading to them, have an unconditional probability table, with a single entry that represents their probability. The “child” nodes, which have one or more arrows leading to them, have a conditional probability table, which indicates the conditional probability of that node, given all possible combinations of the presence or absence of its parent nodes.

**FIGURE 1:**
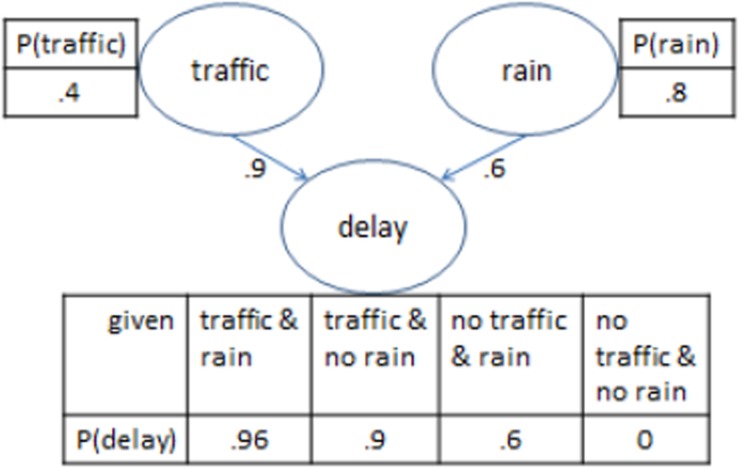
Illustration of the components of a Bayesian network. See main text for details.

Figure 1 provides an example of a simple BN with three nodes, representing two causes that have a potential effect in common. In the figure, the presence of a delay is a function of the (inclusive) disjunction of two mutually independent causes, traffic and/or rain. There is a 40% probability of traffic (which when present on its own, leads to a delay in 90% of cases), and an 80% probability of rain (which when present on its own, leads to a delay in 60% of cases). The numbers in the example assume there are no unknown causes that could lead to a delay in the absence of both traffic and rain.

Once a network is built, it can be queried to assess for example what happens to the probability of a delay if an intervention is made to avoid traffic (such as traveling at a different time of the day).

Bayesian networks are finding increasing use in applied domains requiring people to make complex predictions and decisions on the basis of a range of uncertain and interconnected factors, ranging from forensic (Smit et al., 2016) over medical (Fenton and Neil, 2010; Constantinou et al., 2016) to meteorological contexts (Boneh et al., 2015). However, until now these methods have largely remained accessible only to experts in Bayesian probability theory or practitioners with extensive training (Nicholson et al., 2011; Smit et al., 2016).

In this study, we assessed to what extent the availability of a software tool to construct BNs with minimal training can help lay people solve complex probabilistic reasoning tasks, as might be faced in a range of real world problem solving situations in everyday and professional settings.

The BN software tool used was adapted from the AgenaRisk software^1^ by Ann Nicholson, Erik Nyberg, Kevin Korb, and colleagues at the Faculty of Information Technology of Monash University, Australia (Nicholson et al., 2020, arXiv preprint available at https://arxiv.org/abs/2003.01207). This BN software tool, called BARD (for Bayesian Reasoning via Delphi), differed from AgenaRisk in three main respects relevant to the present study. (a) At the time of the study it implemented only a subset of the functionality of AgenaRisk. (b) The interface was structured in a different way, encouraging a workflow in which users first think of the variables relevant for a problem at hand, and then connect the variables to one another to form a causal network. Next users define the probability tables for each node in the network. Finally, users experiment with or “query” the network to obtain information from it, e.g., by setting one or more nodes to a particular value and assessing what impact this has on the values of the remaining nodes. (c) The software had an inbuilt training module featuring text and short videos, as well as inbuilt pointers to the functionality of each software element that could be accessed throughout the modeling process. The BARD software as a whole also includes features for people to build BNs collaboratively in groups, but we used a version of it, SoloBARD, for which the group related functionality was removed to focus on testing the usefulness of the software for individuals.

## Hypotheses

We tested whether using the BARD software and training system for constructing BNs improves the ability of individuals to solve complex probabilistic reasoning problems, compared to a control group receiving only generic training in probabilistic reasoning. This research question was assessed through the following two hypotheses.

1. The treatment group using the BN software tool will produce higher proportions of correct responses than the control group, measured using predefined rubrics for each problem. The overall score in the rubrics was a composite based on marks awarded for responding to the questions explicitly asked for in the problem statement, alongside marks for providing background information about the problem, such as on the reliability and independence of sources, as well as for providing explanations for the responses given to the explicit questions. This hypothesis was assessed through the computation of effect sizes and confidence intervals.
2. The treatment group will produce higher proportions of correct responses than the control group in the section of the rubrics concerned with probability questions explicitly asked about in the problem statements. This hypothesis was also assessed through the computation of effect sizes and confidence intervals.

## Method

The study was preregistered with the Open Science Framework (OSF). The data, materials and analysis script can be found under: https://osf.io/28w9e/?view_only=d31e21706e4241839e27ea0dff51c98c

### Participants

An initial sample of 72 participants was recruited from the participant recruitment pool of University College London, with 36 in the treatment and control groups, respectively. After accounting for some cancelations, the final sample consisted of 59 participants, 29 in the treatment and 30 in the control group. Participation was remunerated with £10 per hour. In addition, bonuses were given to the highest performing individuals in each group, with £250 to the single top scoring person, £100 to the top tenth percentile, £50 to the next tenth percentile and £25 to the next tenth percentile. All participants were residents of the United Kingdom and had not participated in a previous pilot study. Their mean age was 26.78 years (range 19–68). All indicated being native speakers of English, and 37 indicated having a Bachelor degree or above.

### Materials

All participants worked through three complex probabilistic reasoning problems. These problems were created with the aim of covering a broad range of probabilistic reasoning features. Previous research suggests these are features that people often find difficult to spontaneously grasp (for examples and discussion see Juslin et al., 2009; Sloman and Lagnado, 2015; Rottman and Hastie, 2016; Rehder and Waldmann, 2017). The problems used in this experiment were “Black Site,” “Cyber Attack,” and “Kernel Error.” These were the same problems as had been used in a pilot study aimed at obtaining an impression of baseline problem difficulty. The problem descriptions and the rubrics used to mark the solutions are included in the OSF repository for the study. The probabilistic features measured by each problem are summarized in Table 1 (for more specific theoretical and empirical background to the problems see Dewitt et al., 2018; Liefgreen et al., 2018; Phillips et al., 2018; Pilditch et al., 2018, 2019).

**TABLE 1:** Features measured by the three problems in the experiment.

	**Black site**	**Kernel error**	**Cyber attack**
**General features**			
Alternative hypothesis comparisons	x	x	x
Source reliability/accuracy	x		x
Conflicting evidence	x	x	x
Uncertainty encapsulation	x	x	x
Belief revision/updating		x	x
Base rates	x	x	x
False positive/negatives	x	x	x
Dependent evidence relations			x
Noisy-or	x	x	
**Problem specific features**			
Explaining away/discounting		x	x
Zero-sum fallacy	x		
Common cause vs. multiple independent explanations		x	

Participants in the treatment group worked through the problems using the Bayesian network tool. Their training materials included guidance on how to identify relevant variables for a problem, formulate hypotheses about causal relationships between the variables, estimate the probability of each variable given the presence or absence of its potential causes, and strategies for querying the network to obtain candidate answers to the problem at hand.

Participants in the control group worked through the problems using blank Word documents, with access to the generic information on reasoning with probabilities that they were given during the training. This information included the advice to not only offer a direct answer to the explicit problem questions, but to also explain how and why this answer was arrived at, including a consideration of the reliability and consistency of the sources of information used to come to a conclusion, how likely this conclusion is considered to be, and what information might be missing which, if it became available, could change the assessment of the conclusion in relation to alternative conclusions that could have been drawn instead. Both groups also received guidance on the meanings of the technical terms “hit rate” and “false alarm rate.”

### Design

The experiment followed a between participants design with one predictor variable: Participants were assigned to either the treatment group (receiving the Bayesian network training and software) or the control group (receiving generic information on reasoning with probabilities and a blank Word document).

There were two dependent variables (DVs): total scores on problem rubrics (includes points awarded e.g., for explaining reasoning steps and justifying conclusions arrived at), and question response scores (includes only points awarded for answers to explicit questions). Both dependent variables were measured as proportions of the maximum attainable marks for a problem.

For the above DVs, the study computed (a) effect sizes and (b) 95% confidence intervals (CIs) around the effect sizes. The above measures were complemented with (c) a linear mixed model analysis with random intercepts for participants. The mixed model was used to compute significance tests and CIs for the mean condition differences.

The method for computing effect sizes was chosen on the basis of whether or not the variances were equal in the treatment and in the control group. Equality of variances was assessed through the Levene test (using the leveneTest function of the car package in R). It was determined that if the test indicated that the variances were equal, then effect sizes would be computed using the Hedges’ g measure for the pooled variance (Hedges’ g is similar to Cohens’ d but it corrects for a bias in the latter). If in contrast, the Levene test indicated that the variances were unequal in the two groups, then effect sizes would be computed using Glass’ delta, a measure designed for situations of unequal variance. The linear mixed model analysis was performed in R (R Core Team, 2017) using the lmer function of the lme4 package (Bates et al., 2015).

Participants were assigned to one of the two groups in a pseudo-random way, based on the study dates for which they signed up. The same study advert was used for all study dates. Participants in both groups worked through the three reasoning problems. The order of presentation of the problems was counterbalanced between participants, so that overall each possible problem order occurred approximately equally likely in both groups.

### Procedure

The testing took place in a computer based lab setting under exam conditions. Participants in the treatment group worked through the problems using the Bayesian network system, and their responses – in the form of written reports – were collected from within the system. Participants in the control condition worked through the problems using blank Word documents.

Each group was tested on two full consecutive days. The testing dates took place on different weeks for the two groups to facilitate blinding. Participants in each group were given 2.5 h. to work through each of the three problems, and they were offered lunch and coffee during the session breaks. No performance feedback was provided to participants in either group.

#### Rater Training

To ensure that participants’ reports were marked in an impartial way, nine raters were recruited from university mailing lists, none of whom were associated with the project. The raters received ~7 h of training. Rater training took place over a single full day. The day was split into four sessions, with the first three corresponding to the three problems administered. Within each problem session, raters first read the problem text and then discussed the problem structure as a group. Following this, raters read the rubric and were able to ask any questions and discuss any potential ambiguous elements as a group. Raters then rated a participant report from a pilot experiment. In the final session, raters rated three further reports, one for each problem, totaling six reports marked over the course of the day.

#### Participant Training

Participants in the control group were given 1.5 h to work through the generic training in reasoning with probabilities, and were able to access the training again at any point during the day. Participants in the treatment group were allowed 3.5 h to work through the training material embedded in the BN software. The difference in training time between groups was due to the experimental group having a wider range of material to work through than the control group. Both groups were given the opportunity to refresh their knowledge of training materials for 30 min on the second day of testing, prior to continuing with the problems.

#### Report Rating

Participant reports from both conditions were marked by the nine independent raters working with the problem specific rubrics. Reports were assigned randomly to raters, and all reports were marked by two different raters, with the mean score across the two raters used as the final variable. We took the mean of the two ratings rather than asking raters to discuss potential discrepancies until reaching an agreement in order to allow rater’s judgments to be based on a larger amount of independent information (Hahn et al., 2019). Interrater reliability^2^ was 0.789 for the treatment group and 0.636 for the control group. Raters were instructed to take ∼30 min to mark each report and were allocated 47 reports to mark each. The raters could not be fully blinded to condition because the two conditions used different templates for their answers. However, the raters were not informed about which template corresponded to which condition, nor of any of the study hypotheses.

## Results and Discussion

The average ratings of the two markers for each participant and problem were converted into proportions of the total attainable scores for each problem, separately for each of the two DVs.

### Total Rubric Score

**FIGURE 2:**
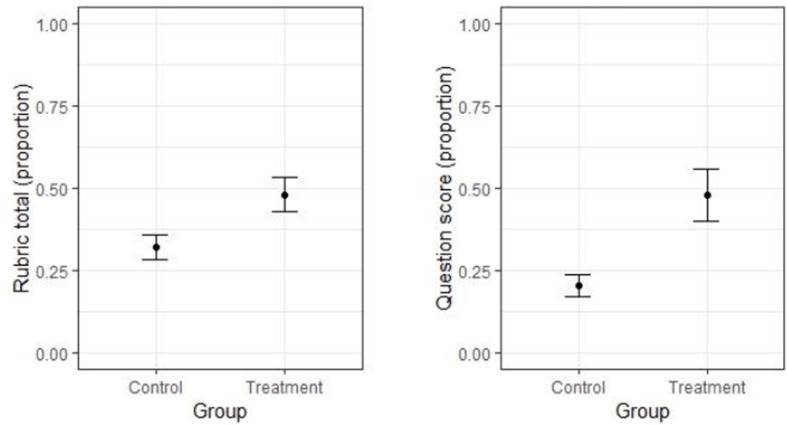
**Left panel:** Means (and their 95% CIs) for the two groups on the total rubric score. **Right panel:** Means (and their 95% CIs) for the two groups on the explicit problem questions.

The average proportion of correct responses based on the total rubric score is shown in the left panel of Figure 2 for each group. The CIs in the figure suggest that the variance was larger in the treatment group than in the control group. The Levene test showed that this difference was significant [*F*_(1, 175)_ = 13.782, *p* < 0.001], thus Glass’ delta rather than Hedge’s g was used as effect size measure. Glass’ delta and the CIs around it were computed using the smd.c and ci.smd.c functions, respectively, both from the MBESS R package. The effect size of the difference between groups on the total rubric scores was large: it reached 0.85 on average, with a 95% CI of [0.527, 1.166].

In accordance with the above results, the linear mixed model indicated that performance in the treatment group (estimated marginal mean = *EMM* = 0.479) was significantly higher than in the control group [*EMM* = 0.321; *t*(57) = 3.546, *p* < 0.001] and inclusion of the predictor for group in the model led to a significant improvement in model fit [*X*^2^(1) = 11.760, *p* < 0.001].

### Explicit Problem Questions

The average proportion of correct responses based on the explicit problem questions is shown in the right panel of Figure 2 for each group. As in the previous analysis, the CIs in the figure suggest that the variance was larger in the treatment group than in the control group. The Levene test showed that this difference was significant [*F*_(1, 175)_ = 46.141, *p* < 0.001], so that Glass’ delta rather than Hedges’ g was used as effect size measure. The effect size for the difference between groups on the explicit question scores was again large, and was numerically larger than that for the total rubric score. It reached 1.62 on average, with a 95% CI of [1.239, 1.996].

In line with the above finding, performance in the treatment group (*EMM* = 0.480) was significantly higher than that in the control group [*EMM* = 0.203; *t*(57) = 4.752, *p* < 0.001], and inclusion of the predictor for group in the model led to a significant improvement of model fit [*X*^2^(1) = 19.691, *p* < 0.001].

Further corroborating analysis carried out separately for each problem can be found in Appendix.

## Conclusion

In this study we investigated whether access to a Bayesian network modeling tool, together with a limited amount of embedded training resources in its use, can help lay people solve complex probabilistic reasoning problems, involving multiple dependencies between uncertain pieces of information that can dynamically change as more information becomes available.

The results were clear cut, providing strong evidence for an advantage in performance of the group with access to the Bayesian network tool compared with the control group having access only to generic training on probabilistic reasoning.

This finding provides a proof of principle that Bayesian network modeling can be made accessible to wider population sectors with minimal, self-directed training. Its introduction in areas such as intelligence analysis, medical or forensic diagnostics, as well as environmental or economic risk forecasting therefore likely constitutes less of an entry burden and uphill task than might be initially thought. Its wider use in these and other domains could bring about substantial benefits to its users given the normativity of the Bayesian framework, which allows people to minimize prediction error in a coherent way, preventing us from getting into situations in which any decision outcome leads to a sure loss (Ramsey, 1926/1990; Pettigrew, 2016; Vineberg, 2016). It can help increase our understanding of the relevant structure of a problem at the same time as the effectiveness with which our concomitant decisions help us to achieve our goals.

## Data Availability Statement

All datasets generated for this study can be found in the Open Science Framework under https://osf.io/28w9e/?view_only=d31e21706e4241839e27ea0dff51c98c.

## Ethics Statement

The studies involving human participants were reviewed and approved by the Research Ethics Committee, Department of Psychological Sciences, Birkbeck, University of London. The patients/participants provided their written informed consent to participate in this study.

## Author Contributions

NC: conceptualization, data curation, formal analysis, investigation, methodology, project administration, visualization, writing – original draft, and writing – review and editing. SCD: investigation, project administration, and writing – review and editing. SD, AL, KP, and MT: conceptualization, investigation, methodology, project administration, and writing – review and editing. UH: conceptualization, formal analysis, funding acquisition, investigation, methodology, project administration, resources, supervision, and writing – review and editing. DL: conceptualization, funding acquisition, investigation, methodology, project administration, resources, supervision, and writing – review and editing. TP: conceptualization, investigation, methodology, project administration, software, and writing – review and editing.

## Disclaimer

The views and conclusions contained herein are those of the authors and should not be interpreted as necessarily representing the official policies, either expressed or implied, of ODNI, IARPA, or the U.S. Government. The U.S. Government is authorized to reproduce and distribute reprints for governmental purposes notwithstanding any copyright annotation therein.

## Conflict of Interest

The authors declare that the research was conducted in the absence of any commercial or financial relationships that could be construed as a potential conflict of interest.

## Figures and Tables

**FIGURE A1:**
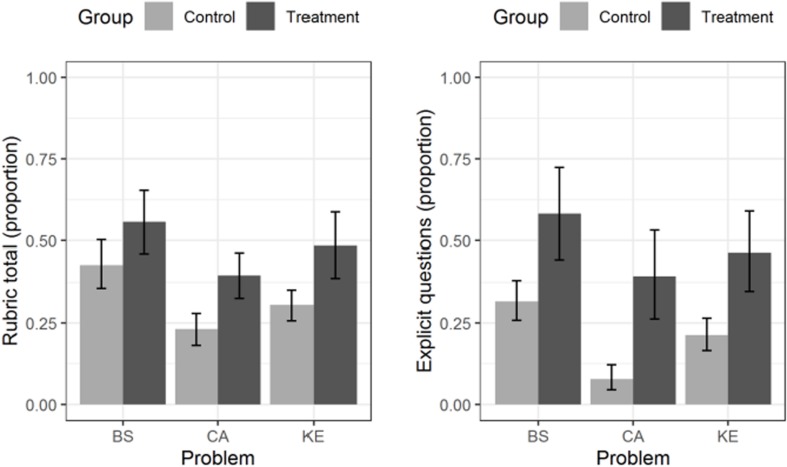
**Left panel:** Means (and their 95% CIs) for the total rubric score in each group, separately for each problem. BS = “Black Site”, CA = “Cyber Attack”, KE = “Kernel Error”. **Right panel:** Means (and their 95% CIs) for the explicit problem questions in each group, separately for the same three problems.

## Footnotes

1 www.agenarisk.com

2 Interrater reliability was measured as intraclass correlation, in a two-way model of type agreement, using the icc function of the irr package in R.

## Appendix

### Additional Exploratory Analysis

We conducted an additional exploratory analysis not included in our preregistration. Its aim was to assess the generalizability of the findings across problems. The left panel of Figure A1 displays the means and 95% confidence intervals of the total rubric score in each group, separately for each of the three problems. The right panel of Figure A1 displays the same information as the left panel, but for the explicit problem questions.

The figures show clearly that for both dependent variables, the higher performance of the treatment group over the control group was not driven only by a subset of the problems used, but held across problems. This was corroborated by a linear mixed model analysis similar to the one reported in the confirmatory section of the results. This analysis showed that the main effect of group was significant not only overall, but also for each problem considered individually (for the total rubric score: lowest *t* = 2.322, highest *p* = 0.022; for the explicit problem questions: lowest *t* = 3.453, highest *p* = 0.0004; adjusted for multiple comparisons using the Sidak procedure).
